# First detection of Hepatitis E virus (*Orthohepevirus C*) in wild brown rats (*Rattus norvegicus*) from Great Britain

**DOI:** 10.1111/zph.12581

**Published:** 2019-04-29

**Authors:** Ellen G. Murphy, Nicola J. Williams, Daisy Jennings, Julian Chantrey, Ranieri Verin, Sylvia Grierson, Lorraine M. McElhinney, Malcolm Bennett

**Affiliations:** ^1^ NIHR Health Protection Research Unit in Emerging Zoonotic Infections Institute of Infection and Global Health, NCZR Neston UK; ^2^ Epidemiology and Population Health Institute of Global Health, NCZR Neston UK; ^3^ Wildlife Zoonoses and Vector Borne Disease Research Group Animal and Plant Health Agency Weybridge UK; ^4^ Department of Veterinary Pathology & Public Health, School of Veterinary Science University of Liverpool Liverpool UK; ^5^ Department of Virology Animal and Plant Health Agency Addlestone UK; ^6^ School of Veterinary Science University of Nottingham Leicestershire UK

**Keywords:** hepatitis E virus, *Orthohepevirus C*, public health, rat, reservoir host, zoonosis

## Abstract

In the United Kingdom, there has been an increase in the number of hepatitis E virus (HEV) infections in people annually since 2010. Most of these are thought to be indigenously acquired *Orthohepevirus A* genotype 3 (HEV G3), which has been linked to pork production and consumption. However, the dominant subgroup circulating in British pigs differs from that which is found in people; therefore, an alternative, potentially zoonotic, source is suspected as a possible cause of these infections. Rodents, brown rats (*Rattus norvegicus*) in particular, have been shown to carry HEV, both the swine HEV G3 genotype and *Orthohepevirus C*, genotype C1 (rat HEV). To investigate the prevalence of HEV in British rodents, liver tissue was taken from 307 rodents collected from pig farms (*n* = 12) and other locations (*n* = 10). The RNA from these samples was extracted and tested using a pan‐HEV nested RT‐PCR. Limited histopathology was also performed. In this study, 8/61 (13%, 95% CI, 5–21) of brown rat livers were positive for HEV RNA. Sequencing of amplicons demonstrated all infections to be rat HEV with 87%–92% nucleotide identity to other rat HEV sequences circulating within Europe and China (224 nt ORF‐1). Lesions and necrosis were observed histologically in 2/3 samples examined. No rat HEV RNA was detected in any other species, and no HEV G3 RNA was detected in any rodent in this study. This is the first reported detection of rat HEV in Great Britain. A human case of rat HEV infection has recently been reported in Asia, suggesting that rat HEV could pose a risk to public health.

**Impacts**

- This article describes a hepatitis E virus (HEV) survey of wild rodents from 12 pig farms and 10 other locations in Great Britain (GB). Rodent liver tissue was screened for HEV RNA using a pan‐HEV ORF 1 RT‐PCR assay.
- The HEV most commonly associated with human infections in GB (*Orthohepevirus A* (HEV G3)) was not detected in any rodent sampled. However, the HEV species associated with rats (*Orthohepevirus C*, rat HEV) was detected, for the first time in GB, in 8/61 (13%, 95% CI, 5–21) of brown rats tested.
- Tests currently used in GB to identify the HEV source of viral hepatitis in humans may not detect rat HEV. Orthohepevirus C infection has been shown to be zoonotic, although its wider risk to public health is still unknown.

## 1 INTRODUCTION

Hepatitis E virus (HEV) is a leading cause of viral hepatitis globally, causing an estimated three million cases of acute hepatitis and 70,000 deaths annually (de Guerra, Kampa, Morsoletto, Junior, & Ivantes, 2017). While most infections are probably self‐limiting and subclinical, infection can lead to liver failure, chronic hepatitis and cirrhosis with a higher mortality rate among pregnant women and the immunocompromised (Guerra et al., 2017). The number of reported human HEV infections in Great Britain (GB) had been increasing annually, from 368 cases reported in 2010 to 1,243 in 2016 in England and Wales (PHE, 2018a), although recent data (2016–2017) show a decreasing trend in the number of hepatitis E cases in England and Wales, with 912 cases reported (PHE, 2018a). HEV RNA has also been detected in 0.04% donated blood products (Hewitt et al., 2014). Most of the reported human HEV infections were due to *Orthohepevirus A* genotype 3 (HEV G3) and were likely zoonotic in origin as HEV G3 has a wide host range and there is a well‐established link between HEV infection and pig meat products in industrialized countries. In GB, HEV has been found in point‐of‐sale British pork sausages (Berto, Martelli, Grierson, & Banks, 2012) and pig liver (Banks et al., 2010). Furthermore, an abattoir study reported that 93% of pigs at slaughter had antibodies to HEV (Grierson et al., 2015), and 21.5% of pig faecal samples and 22% of slurry lagoons were positive for HEV RNA on pig farms (McCreary et al., 2008). However, while the genotype of HEV circulating in people in the UK is HEV G3 subgroup 2, HEV in British pigs is mainly HEV G3 subgroup 1 (Ijaz et al., 2014). Thus, most human infections in the UK appear to be either from imported pork products (Said et al., 2017; Salines, Andraud, & Rose, 2017) or there may be an alternative zoonotic source such as peri‐domestic wildlife.

Rodents have been shown to be susceptible to infection with a diverse range of HEV species and could, therefore, be potential sources of human and livestock (including pig) infections (Ryll et al., 2017; Takahashi et al., 2014). Large populations of some rodent species are common on and around farms due to the availability of food and shelter. In a study in Japan, 10/56 brown rats (*Rattus norvegicus*) were positive for HEV, which had 95.2%–100% genetic similarity to the swine HEV isolated from pigs on the same farm (Kanai et al., 2012). In the UK, HEV G3 RNA subgroup 1 has been reported in the intestines (not livers) of house mice (*Mus musculus*) on one pig farm, but this is believed to be due to ingestion of virus from pig excreta rather than the mice being infected (Grierson, Rabie, Lambert, Choudhury, & Smith, 2018). Furthermore, brown rats have been shown to have their own distinct species of HEV, *Orthohepevirus C*, genotype C1 (rat HEV). Rat HEV was first detected in Germany in 2010 (Johne et al., 2010) and since then has been identified in the USA, China, Vietnam and continental Europe (Ryll et al., 2017). Prior to this study, rat HEV had not been detected in the UK (Grierson et al., 2018). Rat HEV was previously not thought to be zoonotic; however, the first case of human infection in a 56‐year‐old man was recently identified in Hong Kong (HKU Med, 2018; Sridhar et al., 2018) and since then other cases of human rat HEV infection reported (Andonov et al., 2019; Fleming, 2018).

## 2 MATERIALS AND METHODS

### 2.1 Rodents

**Figure 1:**
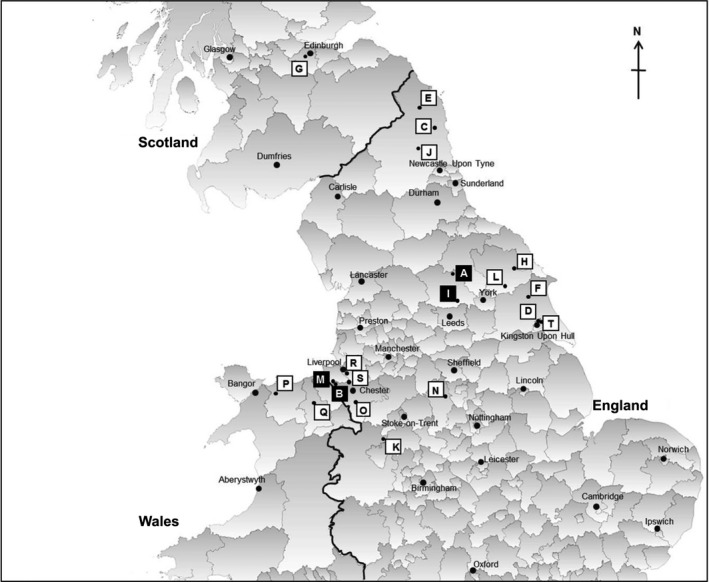
Map indicating the locations of the sites sampled in this study. The site I.D.s correspond to those shown in Table 1. The black squares ■ indicate the sites where HEV‐positive rats were detected (sites A, B, I, M). This map was created using GQIS Desktop V.3.2.3 software

The aim of this study was to investigate HEV infection in British rodents. Peri‐domestic rodents (*n* = 307) were collected from twelve pig farms and ten non‐pig farm locations across Northern England, Wales and Scotland in 2014–2016 (Figure 1). Rodents were also donated from forestry sites, pest control programmes or collected as roadkill. Humane killing of live‐caught rodents and a post‐mortem examination were performed. The prevalence of HEV G3 in the pigs on the pig farms was unknown.

### 2.2 Pan‐HEV ORF1 RT‐PCR assay

RNA was extracted from liver tissue using GenElute Mammalian Total RNA Mini‐prep kit (Sigma‐Aldrich) and cDNA synthesized using RevertAid RT Reverse Transcription Kit (Thermo Fisher Scientific), both according to manufacturers' instructions. A previously published pan‐HEV ORF1 RT‐PCR (Johne et al., 2010) was adapted and performed using 5x HOT FIREPol^®^ Blend Master Mix with 15 mM MgCl_2_ (Solis BioDyne) with primers HEV‐cs and HEV‐cas (Johne et al., 2010) at 95°C for 15 min followed by 40 cycles of 94°C for 30 s, 55°C for 30 s, 74°C for 45 s with a final elongation of 74°C for 5 min. This was followed by a second round with primers HEV‐csn and HEV‐casn (Johne et al., 2010) at 95°C for 15 min followed by 35 cycles of 94°C for 30 s, 62°C for 30 s, 72°C for 45 s and a final elongation of 72°C for 7 min. Positive controls for HEV G3 and rat HEV were used in the optimization of in this assay. Products were visualized on a 2% agarose gel under UV light after gel electrophoresis at 120V for 65 min.

### 2.3 Sequence analysis

PCR amplicons were sequenced and analysed in MEGA 7 (Kumar, Stecher, & Tamura, 2016). Sequences were aligned with published BLAST partial sequences of *Orthohepevirus C1*. A phylogenetic tree was constructed using a best fit model of Kimura 2‐parameter (Kimura, 1980) and bootstrap analysis of 1,000 repeats.

### 2.4 Real‐time PCR assays

To confirm rat HEV, in the absence of sequence data, two published rat HEV‐specific real‐time PCR assays were used (Johne et al., 2012; Mulyanto et al., 2014), with Superscript™ III Platinum™ One‐Step qRT‐PCR kit (Invitrogen) and the published primers and probes(Johne et al., 2012; Mulyanto et al., 2014). Cycling parameters for both assays were 50°C for 15 min followed by 95°C for 2 min, then 45 cycles of 95°C 15 s and 59°C for the Mulyanto et al. (2014) assay or 55°C for Johne et al. (2012) assay, each for 30 s.

### 2.5 Histology

A subset of liver samples were fixed in 10% buffered formalin, and three samples (R1, R5 and R43), that tested HEV RNA‐positive, were paraffin‐embedded and examined microscopically. Haematoxylin and eosin (H&E) staining and immunohistochemistry were performed to characterize morphologically any hepatic lesions and to study the phenotype of lymphocytic infiltrates, respectively. To characterize T and B lymphocytic phenotypes in one rat (R5) liver with prominent lesions, commercial antibodies were used following manufacturer's instructions: Dako polyclonal rabbit anti‐CD3 clone 0452, AbD Serotec monoclonal mouse anti‐CD79a clone HM57 and BD Laboratories monoclonal mouse anti‐PAX‐5 clone 610863.

## 3 RESULTS

HEV RNA was detected only in the livers of brown rats (8/61, 13%, 95% CI 5–21; Table 1). Positive rats were detected at four separate locations, which included three pig farms (sites A, B and I) and a dairy farm (site M).

**Table 1:** Results obtained using the pan‐HEV ORF1 RT‐PCR assay to detect the presence of HEV RNA in liver tissue

Rodent species	Site type	Map I.D.	GB region	PCR result	Species total
*Rattus norvegicus*	Pig farm	A	North Yorkshire	2/16	8/61 (13%, 95% CI 5–21)
	Pig farm	B	Cheshire	1/10	
	Pig farm	D	East Yorkshire	0/1	
	Pig farm	F	East Yorkshire	0/2	
	Pig farm	H	North Yorkshire	0/1	
	Pig farm	I	West Yorkshire	2/16	
	Dairy farm	M	Cheshire	3/5	
	Beef farm	N	Derbyshire	0/1	
	Smallholding	Q	Denbighshire	0/1	
	Commercial	R	Merseyside	0/3	
	Residential	S	Cheshire	0/4	
	Road	T	East Yorkshire	0/1	
*Mus musculus*	Pig farm	A	North Yorkshire	0/1	0/97
	Pig farm	C	Northumberland	0/38	
	Pig farm	D	East Yorkshire	0/9	
	Pig farm	E	Northumberland	0/16	
	Pig farm	F	East Yorkshire	0/3	
	Pig farm	G	Midlothian	0/6	
	Pig farm	H	North Yorkshire	0/3	
	Pig farm	K	Shropshire	0/6	
	Pig farm	L	North Yorkshire	0/15	
*Apodemus sylvaticus*	Pig farm	A	North Yorkshire	0/1	0/48
	Pig farm	F	East Yorkshire	0/10	
	Pig farm	G	Midlothian	0/9	
	Pig farm	I	West Yorkshire	0/2	
	Pig farm	J	Northumberland	0/20	
	Dairy farm	M	Cheshire	0/6	
*Microtus agrestis*	Pig farm	H	North Yorkshire	0/1	0/19
	Beef farm	O	Cheshire	0/2	
	Rural	P	Caernarfonshire	0/16	
*Myodes glareolus*	Pig farm	A	North Yorkshire	0/7	0/49
	Pig farm	D	East Yorkshire	0/1	
	Pig farm	E	Northumberland	0/8	
	Pig farm	F	East Yorkshire	0/12	
	Pig farm	H	North Yorkshire	0/9	
	Pig farm	I	West Yorkshire	0/4	
	Pig farm	J	Northumberland	0/3	
	Dairy farm	M	Cheshire	0/1	
	Beef farm	O	Cheshire	0/3	
	Rural	P	Caernarfonshire	0/1	
*Sciurus vulgaris*	Forest 1	n/a	North Wales (various)	0/21	0/21
*Sciurus carolinensis*	Forest 2	n/a	Merseyside (various)	0/12	0/12

Note

The site type and county locations for where each sample was obtained are shown. The map I.D.s correspond to Figure 1.

Associations between HEV infection with age (6/43, 14% of adults and 2/18, 11% juveniles were HEV+) or sex (7/39, 18% of males and 1/22, 4% of females were HEV+) of the rats were tested through Fisher's exact test. No association with age (*p* = 1) or sex (*p* = 0.24) was found to be statistically significant, although this could be due to the small sample size.

**Figure 2:**
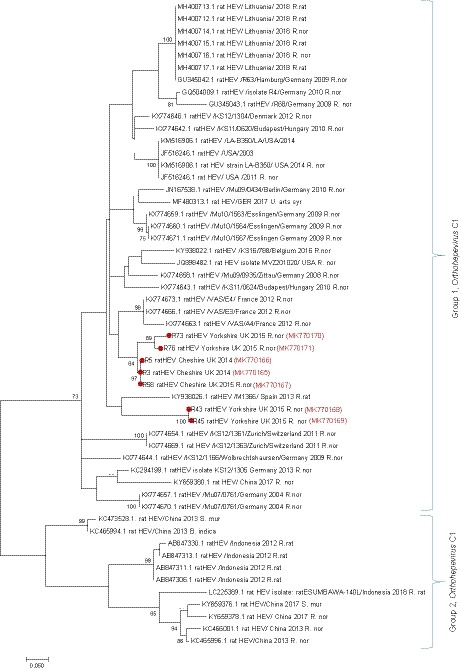
Phylogenetic tree of amplicon sequences from this and other published studies. The tree was inferred using the maximum‐likelihood method using Kimura 2‐parameter model (Kimura, 1980) constructed from 53 nucleotide sequences with 224 positions in the final data set of a partial ORF‐1 fragment of Orthohepevirus C, genotype C1. These included 46 published sequences and seven sequences from this study (indicated by ●). Phylogenetic analyses were conducted in MEGA7 (Kumar et al., 2016). Species abbreviations; *R. nor* (*Rattus norvegicus*), *R. rat* (*Rattus rattus*), *B. indica* (*Bandicota indica*), *S. mur* (*Suncus murinus*) and *U. arts syr* (*Ursus arctos syriacus*). Bootstrap values of >70 are shown [Colour figure can be viewed at wileyonlinelibrary.com]

**Figure 3:**
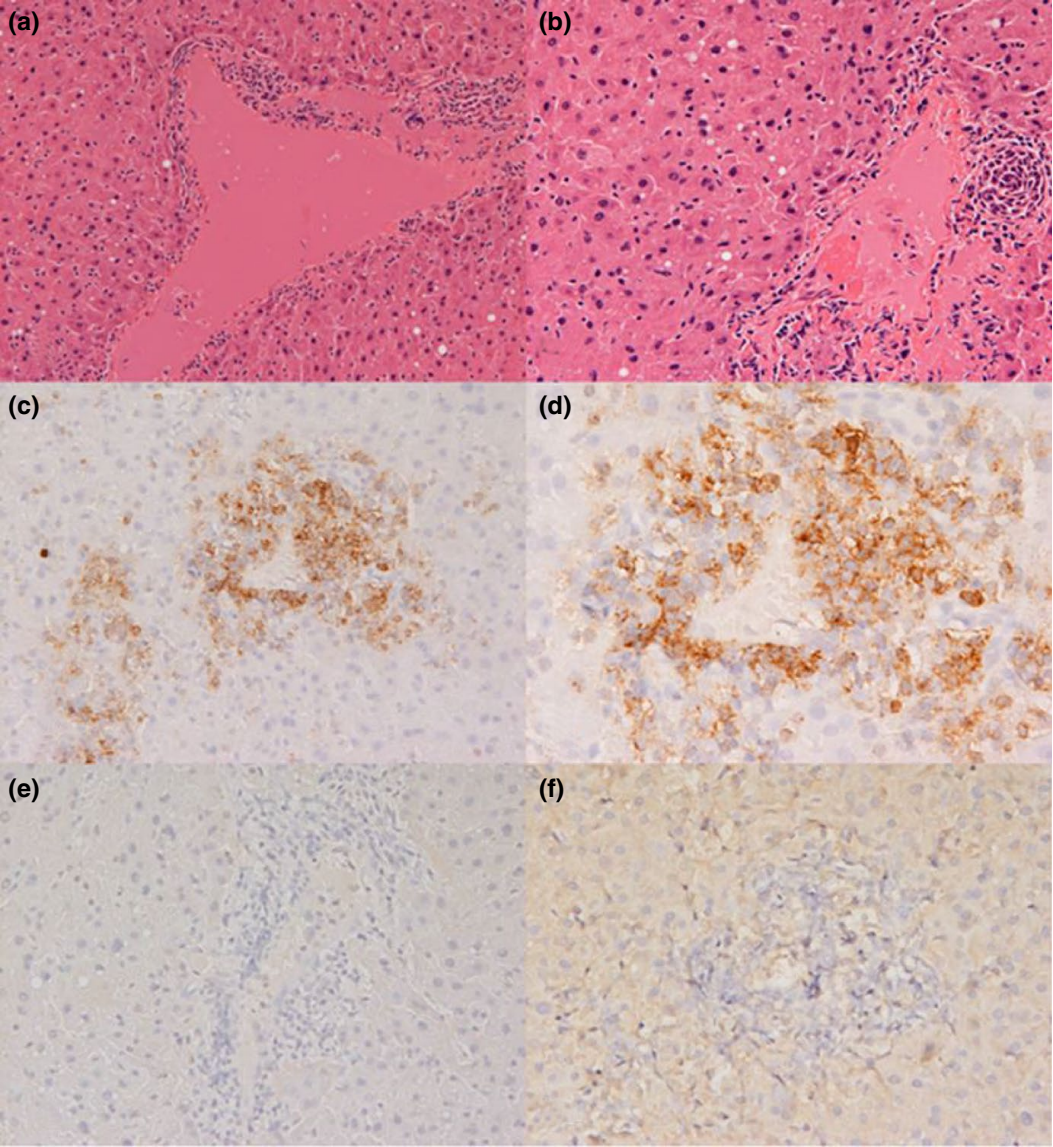
Histology and immunohistochemistry (IHC) of liver from rat R5. (a and b) multifocal mild lymphocytic periportal infiltrates, stained H&E. (c and d) different magnifications of the same portal area showing dense lymphocytic infiltrates composed of CD3‐positive small round cells (T lymphocytes), CD3 IHC. (e) adjacent microscopic field to that in d and e and showing the absence of B lymphocytes in the periportal infiltrates, CD79a IHC. (f) adjacent microscopic field that in d and e showing the absence of B lymphocytes in the periportal infiltrates, PAX‐5 IHC [Colour figure can be viewed at wileyonlinelibrary.com]

Sequence analysis showed that all PCR amplicons were *Orthohepevirus C,* genotype C1 (rat HEV). Sequence data (224 nt) were not obtained for one rat but were confirmed to be rat HEV by testing using primers and probe from two published real‐time PCRs for the specific detection of rat HEV (Johne et al., 2012 [Ct 34.12]; Mulyanto et al., 2014 [Ct 33.29]). The sequences in this study (GenBank accession numbers MK770165, MK770166, MK770167, MK770168, MK770169, MK770170 and MK770171), although not identical, were shown to have a relatively high level of genetic similarity (87%–92% nt identity) with rat HEV sequences from mainland Europe and some sequences from China (Figure 2). No HEV‐G3 RNA was detected in these rats or any other rodents in this study. Histological examination of two of three rat HEV‐positive livers revealed multifocal hepatocellular necrosis. One rat liver was observed to have periportal lymphocytic inflammation, which immunohistochemistry demonstrated to be mostly CD3 positive and CD79a and PAX‐5 negative (Figure 3).

## 4 DISCUSSION

In this study, no HEV G3 RNA was detected in any of the 307 rodent livers tested, although *Orthohepevirus C*, genotype C1 (rat HEV) RNA was detected in 8/61 (13%, 95% CI 5–21) brown rats. Previously, HEV G3 RNA was detected in the intestinal contents, but not liver, of four house mice from one British pig farm and interpreted as the virus passing through the gut from ingested faecal matter rather than infecting the mice (Grierson et al., 2018). The low prevalence in that study and lack of HEV G3 in any rodents tested in this study supports the argument that mice are only accidental hosts of HEV G3 in the UK, and rodents may not have a significant role in the transmission and maintenance of HEV G3 in British pig herds, nor do they pose a significant zoonotic risk, at least in terms of HEV G3 infections.

However, there may be strain differences in the infectivity of HEV G3 to rodents, including rats. Japanese (Kanai et al., 2012) and US (Lack, Volk, & Bussche, 2012) studies have reported HEV G3 RNA in the liver tissue of wild rats (*R. norvegicus* and *Rattus rattus*), while experimental studies found laboratory rats were not susceptible to infection (Li, Ami, Suzaki, Takeda, & Takaji, 2013). In this study, *Orthohepevirus C*, genotype C1 (rat HEV) RNA was detected in British rats, and sequence analysis showed it to be closely related to those found in other European countries and China. The brown rat originated in Central Asia and has only been present in Europe, including the UK, for around 300 years (Harris & Yalden, 2008), probably through multiple migrations and introductions. Hence, it is perhaps not surprising that, if rat HEV is endemic in brown rats, sequences from British rats should be so similar to those from the rest of Europe (Figure 2).

Rat HEV is readily transmissible between rats (Johne et al., 2010) and has been detected in several species of the *Rattus* genus, including the black rat (*R. rattus*; Ryll et al., 2017), Tanezumi rat (*Rattus tanezumi*) and *Rattus rattoides losea* (Li et al., 2013). In addition, strains of the C1 genotype have also been detected in a variety of species outside the *Rattus* genus, such as in greater bandicoot rats (*Bandicota indica*; Li et al., 2013), the Asian musk shrew (*Suncus murinus*; Guan et al., 2013; Li et al., 2013) and a Syrian brown bear (*Ursus arctos syriacus*) from a German zoo (Spahr et al., 2017). However, experimental cross‐species infection has been attempted in laboratory mice (Debing et al., 2016) and pigs (Cossaboom et al., 2012) with no success.

Apart from the single reported case in Hong Kong (HKU Med, 2018), rat HEV has not been detected in humans (Doceul, Bagdassarian, Demange, & Pavio, 2016) and is not readily transmitted to non‐human primates (Purcell et al., 2011). Apart from a serological study in Germany which found that forestry workers had antibody more reactive to rat HEV antigens than to other HEV genotypes (Dremsek et al., 2012), and the successful replication of this virus in human hepatoma cells (Jirintai et al., 2014), there is no compelling evidence that rat HEV poses a significant risk to human. However, the current PCR and serological (IgG, IgM, Ag) diagnostic tests commonly used by public health laboratories (PHE, 2018b) may not optimally detect rat HEV, even if a person was infected. The Wantaï HEV detection ELISA assay, which is commonly used in the UK for diagnosing HEV G3 infections, was designed to detect antibody responses to *Orhtohepevirus A* (Genotype 1–4) (Trémeaux et al., 2016). A review of uncharacterized viral hepatitis cases using tests capable of detecting rat HEV may be warranted to determine the true prevalence of this virus in humans.

This is the first report of *Orthohepevirus C*, genotype C1, in Great Britain, and provides further evidence that this virus is both widespread and endemic in the brown rat (*R. norvegicus*). No other HEV species were detected in the British wild rodents sampled in this study, on pig farms or elsewhere, suggesting that wild rodents do not currently pose a significant disease risk, in terms of HEV, to public or livestock health in GB. Immunohistochemical analysis of rat HEV infected brown rat livers showed periportal lymphocytic infiltrates which suggests a T‐lymphocyte rich inflammatory response, as is also described in human HEV infection (Drebber et al., 2013). This could imply a T‐cell immune‐mediated pathogenesis. The histopathologic lesions observed with natural *Orthohepevirus C* infection suggest that rats might make a useful model for studying HEV pathogenesis.

## CONFLICT OF INTEREST

No conflict of interest.

## AUTHORS' CONTRIBUTION

EM, MB, NJW, JC and LM conceived and designed the experiments. EM collected the samples. EM and DJ performed the experiments. EM, MB, NJW, DJ, RV, LM and SG collated, analysed and interpreted the data. All authors contributed to and approved the final manuscript.

## ETHICAL STATEMENT

The University of Liverpool, Veterinary Research Ethics committee approved this study.
